# Neuroanatomical substrate of chronic psychosis in epilepsy: an MRI study

**DOI:** 10.1007/s11682-019-00044-4

**Published:** 2019-02-08

**Authors:** Noriaki Hirakawa, Hironori Kuga, Yoji Hirano, Jinya Sato, Naoya Oribe, Itta Nakamura, Shogo Hirano, Takefumi Ueno, Yuko Oda, Osamu Togao, Akio Hiwatashi, Hiroshi Honda, Shigenobu Kanba, Toshiaki Onitsuka

**Affiliations:** 1 Department of Neuropsychiatry, Graduate School of Medical Sciences, Kyushu University, 3-1-1 Maidashi, Higashiku, Fukuoka, 812-8582 Japan; 2 Johns Hopkins Bloomberg School of Public Health, Baltimore, MD USA; 3 Division of Clinical Research, National Hospital Organization, Hizen Psychiatric Center, Saga, Japan; 4 Department of Clinical Radiology, Graduate School of Medical Sciences, Kyushu University, Fukuoka, Japan

**Keywords:** Chronic interictal epileptic psychosis, MRI, Left postcentral gyrus, Left supra marginal gyrus

## Abstract

There may be different neural bases between subjects with epilepsy only (EP) and interictal chronic epilepsy psychosis (EPS). However, there have been few structural MRI studies of EPS. The current study was conducted to investigate the neural substrate of EPS. T1-weighted images were analyzed in 14 patients with EPS and 14 strictly-matched patients with EP. We conducted volume comparison in the whole brain using voxel-based morphometry (VBM). The VBM method revealed that EPS patients exhibited significantly reduced gray matter volumes in the left postcentral gyrus and the left supra marginal gyrus compared with EP patients (adjusted *p* = 0.029, FDR corrected *q*; *k* = 319 voxels). For clinical correlations, there were no significant associations between psychotic symptoms and gray matter volumes in the left postcentral gyrus and the left supra marginal gyrus. VBM analysis revealed that reduced gray matter volumes in the left postcentral gyrus and the left supra marginal gyrus may be crucial regions for EPS.

## Introduction

The psychoses of epilepsy can be categorized by their temporal relationships with seizures, commonly referred to as ictal, postictal and interictal psychoses. Chronic interictal epileptic psychosis (EPS) subjects show delusional symptoms similar to those seen in patients with schizophrenia, although thought disorders are rare and disintegration of mental boundaries is usually absent in EPS (Nadkarni et al. 2007). On the one hand, EPS subjects have been reported to show less negative symptoms and social anhedonia compared with patients with schizophrenia (Tadokoro et al. 2007). Applying the Diagnosis and Statistical Manual of Mental Disorders to cases of EPS results in diagnoses of “other specified mental disorder due to another medical condition”. However, the mechanism for generation of chronic psychosis with epilepsy subjects is unclear. Investigating the neural basis of EPS is an important research issue; however little examination has received to date.

High resolution magnetic resonance imaging (MRI) is a suitable method for detecting subtle structural differences in the brain. One of the gold standards of MRI research is a neuroanatomically defined and manually delineated Regions of Interest (ROI) method. Another is a voxel-based morphometric (VBM) method to investigate whole brain without a specific hypothesis. In terms of psychoses of epilepsy including EPS, ictal and postictal psychosis, there have been few structural MRI studies, with mixed findings. For the ROI method, Marsh et al. (2001) examined MR images of patients with schizophrenia, epilepsy only (EP), psychoses of epilepsy and healthy control subjects. They reported that all patient groups exhibited ventricular enlargement and reduced temporal lobe, frontoparietal region, and superior temporal gyrus gray matter volumes. Among these groups, the extent of these abnormalities was greatest in patients with psychoses of epilepsy. Tebartz van Elst et al. (2002) reported significant enlargement of bilateral amygdala in psychoses of epilepsy compared with EP patients and healthy control subjects. For the VBM analysis, two studies have reported no significant differences between psychoses of epilepsy and EP (Rusch et al. 2004; Sone et al. 2016). One study has found significant bilateral volume reductions in the inferior, middle and superior temporal gyri and fusiform gyri, and unilateral volume reductions in the left parahippocampal gyrus and hippocampus of patients with psychoses of epilepsy (Sundram et al. 2010). Therefore, structural MRI findings in psychoses of epilepsy still remain uncertain.

In the current study, volume comparison in the whole brain was explored using VBM to compare the EP and EPS groups to investigate the mechanism of generating chronic psychosis in epilepsy patients. In a typical psychosis such as schizophrenia, various gray matter volume reductions have been reported (e.g., Shenton et al. 2001). Therefore, we hypothesized that individuals with EPS will show decreased gray matter volumes somewhere within their brain when compared with EP subjects.

## Materials and methods

### Subjects

**Fig. 1:**
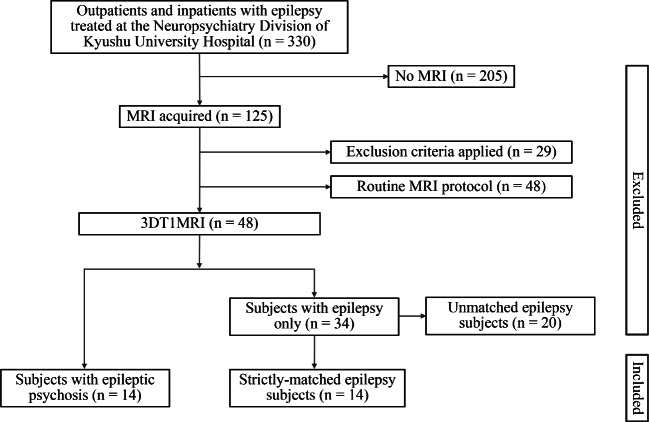
The flowchart of selection of subjects

We assessed inpatients and outpatients diagnosed with epilepsy at the Neuropsychiatry Division of Kyushu University Hospital between 2007 and 2016 (*n* = 330). Two trained neuropsychiatrists (TO and SH) examined seizure type, electroencephalogram (EEG) and neuroimaging (MRI or computed tomography) data. All patients were diagnosed with epilepsy only or epilepsy with mental disorders due to general medical condition using the ICD-10 based on clinical interviews and medical records. A flowchart of the subject selection process is shown in Fig. 1.

Exclusion criteria for participation were: individuals under 16 years or over 65 years old or with unclassified seizures, history of surgery treatment for epilepsy, other psychiatric/neurological illnesses, alcohol or drug abuse or no ability to provide informed consent, as judged by two neuropsychiatrists. All participants gave written informed consent after being given a complete description of the study, which was approved by the Kyushu University Institutional Review Board for Clinical Trials. The present study matched demographic data, epilepsy syndrome classification and focus estimated by interictal EEG and/or clinical symptoms between groups as possible. Finally, MR images were analyzed in 14 patients with EPS and 14 strictly-matched patients with EP. Demographic and clinical data for all subjects are presented in Table 1.

**Table 1:** Demographic and clinical characteristics of the participants

	Epilepsy with psychosis	Matched Epilepsy	χ2 or *t*	*df*	*p*
Male/Female	5/9	5/9	1.00	1	1.00
Age (years)	37.1 ± 9.6	36.5 ± 10.4	0.17	26	0.87
Handedness (right/left)	15/1	15/1	1.00	1	1.00
SES	4.5 ± 0.9	3.6 ± 1.2	2.24	26	0.03*
Parental SES	3.1 ± 0.8	3.4 ± 0.6	−1.03	26	0.32
Education (years)	12.9 ± 2.4	12.3 ± 2.0	0.76	26	0.45
Seizure onset (years)	13.1 ± 8.3	13.0 ± 10.7	0.01	26	0.99
PANSS positive	18.9 ± 6.4	7.6 ± 1.5	6.44	26	<0.001
PANSS negative	13.2 ± 6.7	7.2 ± 0.6	3.34	26	0.003
PANSS total	72.0 ± 18.2	34.9 ± 5.5	7.29	26	<0.001

*Epileptic psychosis group showed significantly lower SES

The socioeconomic status (SES) of the subjects and the parental SES were measured using the Hollingshead two-factor index (Hollingshead 1965). The Positive and Negative Syndrome Scale (PANSS) (Kay et al. 1987) was administered to assess the severity of psychiatric symptoms at the time of scanning. Detailed clinical characteristics of EP and EPS groups are presented in Tables 2 and 3. EPS and EP subjects were taking anticonvulsants and/or antipsychotics in the dosage listed at the time being scanned (see Tables 2 and 3).

**Table 2:** Clinical characteristics of epilepsy groups

				Epilepsy with psychosis					Matched epilepsy
No	Gender	Age	Seizure Type	Focus	Antipsychotic medication	gender	age	Seizure Type	Focus
1	M	45	partial	bilateral temporal	Risperidone 6 mg	M	47	partial	probably bilateral temporal
2	M	37	partial	bilateral temporal	Haloperidol 9 mg	M	44	partial	right temporal
3	F	37	partial	bilateral temporal	Olanzapine 20 mg	F	38	partial	bilateral temporal
4	F	61	partial	bilateral temporal	none	F	58	partial	probably bilateral temporal
5	F	39	partial	bilateral temporal	Quetiapine 50 mg	F	35	partial	bilateral temporal
6	M	35	partial	left temporal	Risperidone 1 mg	M	34	partial	bilateral temporal
7	F	40	partial	right temporal	none	F	42	partial	right temporal
8	F	39	partial	right temporal	Olanzapine 5 mg	F	38	partial	bilateral temporal
9	F	43	partial	right temporal	Propericiazine 25 mg	F	44	partial	right temporal
10	F	37	partial	right temporal	none	F	33	partial	bilateral temporal
11	F	20	partial	unknown	none	F	17	partial	unknown
12	M	30	partial	probably bilateral temporal	Olanzapine 5 mg	M	28	partial	left temporal
13	F	31	generalized		none	F	30	generalized	
14	M	26	generalized		Quetiapine 300 mg, Propericiazine 50 mg	M	23	generalized

**Table 3:** Clinical characteristics of epilepsy groups (cont’d)

No	Epilepsy with psychosis		Matched epilepsy
	Antiepileptics (Daily dose, mg)	main psychotic symptoms	Antiepileptics (Daily dose, mg)
1	VPA 600, CBZ 600, PHT 300	delusion of persecution	CBZ 700, PHT 275, CLB 20
2	CBZ 700, CZP 2.5, CLB 10	reference delusion	VPA 600, PHT 300, LEV 2000
3	CBZ 600, PHT 180, PB 30	delusion of persecution	PHT 200, PB 90
4	CBZ 500, PHT 200, CLB 20	delusion of persecution	PHT 100, ZNS 200, CZP 4
5	VPA 1000, GBP 800	delusion of persecution	PHT 300, CZP 2.5, CLB 10, GBP 1200, LTG 200
6	VPA 1800, CBZ 600	delusion of possession, visual hallucinations	CBZ 400
7	TPM 300, LTG 225, LEV 1500	reference delusion	VPA 400, CBZ 400
8	VPA 600, CBZ 100	delusion of persecution	CBZ 800, CLB 10
9	CBZ 600, PHT 270	reference delusion	CBZ 1100, PHT 200, CLB 10
10	CBZ 750, TPM 350	delusion of persecution	CBZ 800
11	none (during observation)	delusion of persecution	CBZ 400, CLB 10, LTG 50
12	VPA 400, ZNS 200	muddle, reference delusion	VPA 1200, CBZ 400
13	VPA 1200, LEV 1000	delusion of persecution	VPA 600, CBZ 600, PB 90
14	VPA 600, CBZ 600, LTG 75	muddle, reference delusion	VPA 400

*VPA* valproate, *CBZ* carbamazepine, *PHT* phenytoin, *ZNS* zonisamide, *PB* phenobarbital, *CZP* clonazepam, *CLB* clobazam, *TPM* topiramate, *GBP* gabapentin, *LTG* lamotrigine, *LEV* levetiracetam

### MRI data acquisition

T1-Weighted MR images were acquired with a 3D turbo field echo sequence using a 3-Tesla scanner (Achieva TX, Philips Healthcare, Best, The Netherlands) at the Department of Radiology, Kyushu University Hospital. The imaging variables were as follows: repetition time = 8.2 msec, echo time = 3.8 msec, flip angle = 8°, field of view = 24 × 24 cm, number of echoes = 1, matrix = 240 × 240, inversion time = 1025.9 msec, number of slices = 190, and slice thickness = 1 mm. Images were aligned using the line between the anterior and posterior commissures (AC-PC) and the sagittal sulcus to correct head tilt.

### VBM preprocessing and analysis

Total gray matter, white matter, and cerebrospinal fluid volumes were calculated using Statistical Parametric Mapping 12 (SPM12, http://www.fil.ion.ucl.ac.uk/spm), and VBM was also performed using SPM12, running with Matlab R2014a (The Math Works Inc., Natick, MA). T1-weighted images were first segmented for gray matter, white matter and cerebrospinal fluid (CSF) sections using tissue probability maps based on the International Consortium of Brain Mapping (ICBM) template for East Asian brains. Subsequently, we performed diffeomorphic anatomical registration through exponentiated lie algebra (DARTEL) in SPM12 for intersubject registration of gray matter images (Ashburner and Friston 2000). The registered images were then smoothed with a Gaussian kernel of 8 mm full-width half-maximum (FWHM), then transformed to Montreal Neurological Institute stereotactic space using affine and nonlinear spatial normalization implemented in SPM12. The total gray matter, white matter and CSF were generated from the VBM analysis. The total intracranial volumes (ICV) were calculated as the sum of gray matter, white matter and CSF volumes.

### Statistical analysis

Whole-brain voxel-wise comparisons of gray matter between the EPS and EP groups were carried out in SPM12 using general linear model analysis, with age, sex and chlorpromazine equivalents (mg) of antipsychotic medication (Stoll 2001; Woods 2003) as covariates. We used adjusted *p* (false discovery rate (FDR) corrected *q*) < 0.05 as the cut-off for statistical significance after correction for multiple comparisons using FDR.

## Results

**Fig. 2:**
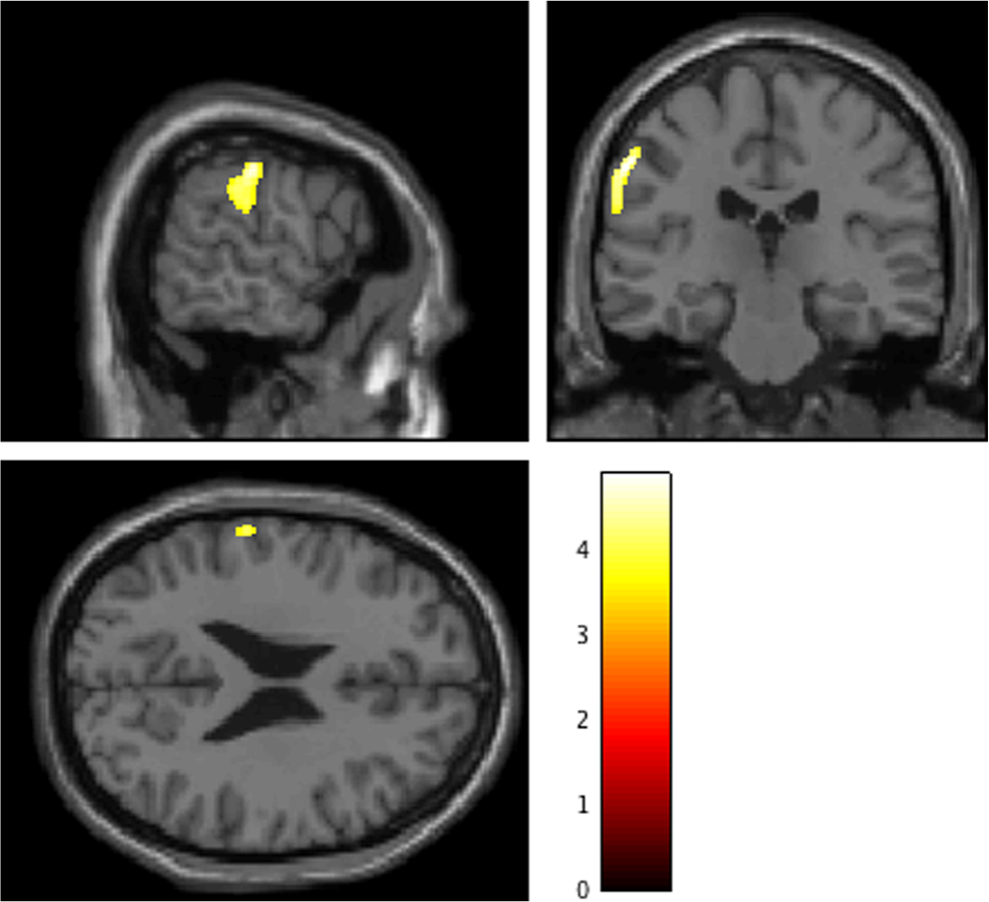
Decreased gray matter regions in EPS patients compared with EP patients. EPS patients exhibited significantly reduced gray matter volumes in the left postcentral gyrus (peak: [−58, −22, 40], *t* = 4.89) and the left supra marginal gyrus (peak: [−62, −27, 32], *t* = 4.41), compared with EP patients. Color bars represent the *t*-value of the contrast (adjusted *p* < 0.05). The [x, y, z] locations indicate Montreal Neurological Institute coordinates

EPS showed significantly lower SES than EP, consistent with reduced functioning due to chronic psychosis (*t*[26] = 2.24, *p* = 0.03). A whole-brain analysis with FDR correction was conducted to examine regional differences in gray matter volume between EPS and EP groups. Patients with EPS exhibited significantly reduced gray matter volumes in the left postcentral gyrus and the left supra marginal gyrus, compared with EP patients (adjusted *p* = 0.029, FDR corrected *q*; *k* = 319 voxels) (see Table 4 and Fig. 2).

**Table 4:** VBM results for anatomical regions, seed voxel coordinates (MNI), *Z*-scores, and cluster sizes for significant group differences (EP > EPS)

Anatomical region	Cluster peak MNI coordinate (mm)			*Z*-score	Cluster size, *k* = (1.5 mm)^3^
	x	y	z		
Left postcentral gyrus	−58	−22	40	3.98	319
Left supra marginal gyrus	−62	−27	32	3.69	

Results are thresholded at adjusted *p* < 0.05, cluster-corrected FDR. MNI: Montreal Neurological Institute

The exploratory analyses were performed in the left postcentral gyrus and the left supra marginal gyrus for the EPS group. There were no significant associations between gray matter volumes and subscale of PANSS scores (−0.16 < *rho* < 0.54; 0.05 < *p* < 0.92).

## Discussion

In the present study, we performed VBM analysis for the whole brain, revealing that EPS patients exhibited significantly reduced gray matter volumes in the left postcentral gyrus and the left supra marginal gyrus compared with EP patients. As noted in the introduction section, some previous MRI studies have conducted whole brain analysis in psychoses of epilepsy, but the results were inconsistent. For example, Rusch et al. (2004) reported no significant cortical gray matter differences between patients with psychoses of epilepsy and those with non-psychotic epilepsy. Sone et al. (2016) also reported no significant differences in morphology between temporal epilepsy patients with and without psychosis. However, Sundram et al. (2010) explored cortical gray matter differences between 10 participants with temporal lobe epilepsy with psychoses and 10 with temporal lobe epilepsy only. They reported significant bilateral volume reductions in the inferior, middle and superior temporal gyri and fusiform gyri, and unilaterally in the left parahippocampal gyrus and hippocampus. They also found significant extratemporal gray matter reduction was found bilaterally in the insula, cerebellum, caudate nuclei and in the right cingulum and left inferior parietal lobule. Sundram et al. used VBM based on non-parametric permutation testing (Bullmore et al. 1999) to examine morphometric differences. However, this testing method is not suitable when the sample includes outliers or covariates, or if the sample size is small. In the present study, we performed VBM comparison of the two groups with three covariates using statistical parametric mapping software (SPM12) with FDR cluster correction. Thus, the gray matter volume reductions we observed in the left postcentral gyrus and the left supra marginal gyrus of EPS patients may be a more robust finding.

In the present study, significant differences were found in the left hemisphere only. It has been reported that patients with schizophrenia show significant gray matter volume reductions in the left superior temporal gyrus but not in the right (e.g., Kasai et al. 2003). Similarly, Fischer et al. (2016) suggested that progressive gray matter reductions of cerebellum and left posterior hemisphere may be involved in delusional development in patients with mild cognitive impairment/Alzheimer’s disease. Thus, left hemisphere gray matter reductions may be associated with generation of psychoses. Moreover, the left supra marginal gyrus contributes to Wernicke’s area, and is a key part of the heteromodal association cortex. Some previous studies have reported left supra marginal gyrus gray matter reduction in patients with schizophrenia (e.g., Buchanan et al. 2004). Recently, Drakesmith et al. (2016) investigated gray matter volumes in non-clinical subjects with and without psychotic experiences. They reported significant left supra marginal gyrus gray matter reductions in subjects with psychotic experiences. The present results revealed that EPS patients exhibited significantly reduced gray matter volumes in the left postcentral gyrus and the left supra marginal gyrus compared with EP patients, suggesting that these regions may be crucial for EPS.

Several significant limitations of the current study are as follows: 1) the sample size was also small as previous studies. Thus, the gray matter reductions we observed in EPS patients should be confirmed with a larger sample. 2) the current study lacked healthy controls or patients with schizophrenia for comparisons. 3) we did not perform a structured clinical interview, and other co-morbid conditions cannot be strictly evaluated. 4) the current study cannot answer the question of whether the volume reduction we observed was associated with progression in the course of illness, whether it is neurodevelopmental in origin, or a combination of both. 5) the current study cannot allow us to exclude the potential effects of chronic medications on gray matter abnormalities in patients.

In conclusion, the present study revealed that reduced gray matter volumes in the left postcentral gyrus and the left supra marginal gyrus, and these regions may be crucial for EPS.
